# C-terminal tail of human immunodeficiency virus gp41: functionally rich and structurally enigmatic

**DOI:** 10.1099/vir.0.046508-0

**Published:** 2013-01

**Authors:** Jonathan D. Steckbeck, Anne-Sophie Kuhlmann, Ronald C. Montelaro

**Affiliations:** 1 Department of Microbiology and Molecular Genetics, University of Pittsburgh School of Medicine, Pittsburgh, PA, 15261, USA; 2 Center for Vaccine Research, University of Pittsburgh, Pittsburgh, PA, 15261, USA

## Abstract

The human immunodeficiency virus (HIV) and acquired immunodeficiency syndrome (AIDS) pandemic is amongst the most important current worldwide public health threats. While much research has been focused on AIDS vaccines that target the surface viral envelope (Env) protein, including gp120 and the gp41 ectodomain, the C-terminal tail (CTT) of gp41 has received relatively little attention. Despite early studies highlighting the immunogenicity of a particular CTT sequence, the CTT has been classically portrayed as a type I membrane protein limited to functioning in Env trafficking and virion incorporation. Recent studies demonstrate, however, that the Env CTT has other important functions. The CTT has been shown to additionally modulate Env ectodomain structure on the cell and virion surface, affect Env reactivity and viral sensitivity to conformation-dependent neutralizing antibodies, and alter cell–cell and virus–cell fusogenicity of Env. This review provides an overview of the Env structure and function with a particular emphasis on the CTT and recent studies that highlight its functionally rich nature.

## Introduction

Human immunodeficiency virus (HIV) is the aetiological agent of acquired immunodeficiency syndrome (AIDS). Despite a significant number of breakthroughs since its discovery in 1983 (Barré-Sinoussi *et al.*, 1983; Gallo *et al.*, 1984), the infection remains pandemic. According to the WHO, 2.7 million people were infected worldwide in 2010 (the last date for which data are available) bringing the total infected population to 34 million individuals, with sub-Saharan Africa being the most affected region with 22.9 million people infected. For nearly three decades, scientists and physicians have been working together to fight the epidemic. Different approaches have been assessed, and some of them have shown significant advances to slow the progression of the disease, such as highly active anti-retroviral therapy. But these strategies, despite successfully increasing the lifespan for treated patients, do not indefinitely halt the progression of the infection. Thus, many efforts have also been made towards the development of an HIV vaccine through the testing of numerous candidates in human clinical trials (for review see McElrath & Haynes, 2010). To date, no or only modest efficacy has been observed. Most of these trials have focused on the HIV-1 envelope as the primary target.

**Fig. 1.**
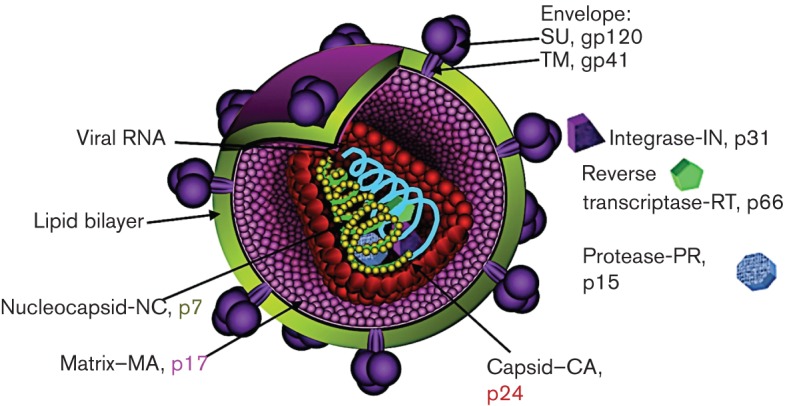
HIV virion structure. Schematic representation of a mature HIV virion detailing the localization of viral proteins and the approximate virion structure. The representation is not to scale.

HIV is a member of the family *Retroviridae*, and belongs to the genus *Lentiviridae*. As a retrovirus, RNA is the genetic material for HIV, and each virion contains two positive-strand copies of the viral genome containing a 5′ cap and a 3′ poly(A) tail, and as such, the viral genome is not infectious (Luciw *et al.*, 2002). Morphologically, HIV is an irregular, roughly spherical virus with a characteristic electron-dense conical core surrounded by a lipid envelope that is derived from the host cell during the budding process (Fig. 1) (Luciw *et al.*, 2002). The virus commonly infects CD4^+^ T-cells, macrophages or dendritic cells. The envelope protein (Env) of the virus is key to the viral infection process as it functions to bind to the receptor and co-receptors at the surface of target cells. Env is the only viral protein exposed on the virion surface, and it is the main target of the host humoral immune system (McElrath & Haynes, 2010). The targeting of Env, through direction of the immune response by vaccination or by traditional small-molecule pharmaceuticals provides arguably the best hope for controlling the epidemic as the goal of these therapies is to prevent the initial infection event (McElrath & Haynes, 2010). As such, the study of Env structure and function has yielded important insights into the properties and features of these viral proteins.

**Fig. 2.**
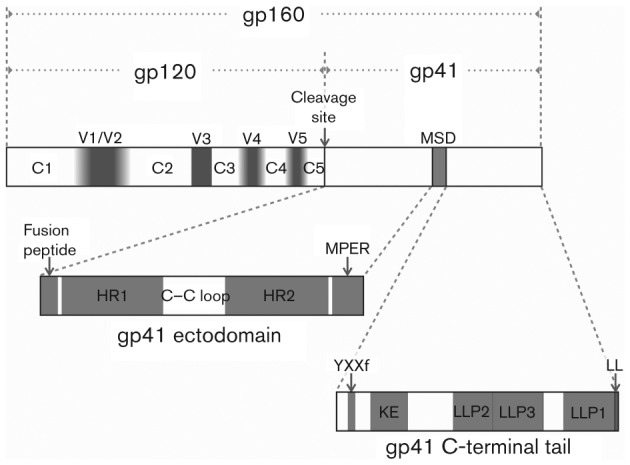
HIV Env primary structure. The Env primary structure is presented schematically, with the immature polyprotein labelled gp160 and the mature, cleaved protein labelled gp120 and gp41. Protein domains are labelled within each mature protein. In gp120 the constant regions are labelled C1–C5 and the variable domains are labelled V1–V5. The shading in the variable regions indicates the relative plasticity of variable region length, with V1/V2 ranging from 50 to 90 aa, V4 from 19 to 44 and V5 from 14 to 36 aa, while V3 does not vary appreciably in size (reviewed by Checkley *et al.*, 2011). In the gp41 ectodomain, the heptad repeat regions (also called the N- and C-heptad repeats) are labelled HR1 and HR2, respectively, and the membrane proximal external region is labelled MPER. In the gp41 CTT, the Kennedy epitope is labelled KE and the lentivirus lytic peptide sequences are labelled LLP1, LLP2 and LLP3. Functional endocytic motifs are labelled YXXΦ (near the N terminus) and LL (at the C terminus).

Functional Env is a trimer of heterodimers derived from extensive processing of the *env* gene product, as presented schematically in Fig. 2. Env is translated and co-translationally *N*-glycosylated as a 160 kDa polyprotein (gp160) in the endoplasmic reticulum (ER). Env gp160 is thought to multimerize in the ER into predominantly trimers before trafficking to the Golgi (Earl *et al.*, 1990, 1991; Pinter *et al.*, 1989). In the Golgi, Env gp160 is cleaved by a cellular furin-like protease at a conserved K/R(X)K/RR motif into gp120 [or surface unit (SU)] and gp41 (or transmembrane) (Freed *et al.*, 1989; McCune *et al.*, 1988). The cleaved gp120 and gp41 products non-covalently associate to form the active trimeric Env unit.

## gp120 structure and function

The primary function of gp120 is to initiate and modulate virus–cell interactions through binding to the primary receptor, CD4, and the co-receptor, predominantly CCR5 or CXCR4. gp120 initiates virus–cell contact by binding to CD4 and subsequently undergoes structural rearrangements that result in the formation of the co-receptor-binding site. These conformational changes allow for high affinity binding of gp120 to co-receptor that in turn leads to conformational changes in gp41 to begin the process of virus–cell membrane fusion (Myszka *et al.*, 2000). Because of its receptor binding functionality and the identification of antibodies that exhibit cross-clade neutralizing activity (broadly neutralizing antibodies), gp120 is a major focus for experimental vaccines (McElrath & Haynes, 2010). In addition, one commercially licensed small molecule therapeutic, maraviroc, targets the gp120–co-receptor-binding interaction (Dragic *et al.*, 2000). However, maraviroc acts by binding CCR-5, and does not specifically interact with gp120 and is inactive against viruses that utilize CXCR4 as co-receptor (Dragic *et al.*, 2000).

gp120 is a structurally complex molecule. Sequence analyses of gp120 from diverse HIV-1 isolates identified a high degree of variation, resulting from the high error rate of reverse transcriptase and biological selection (Preston *et al.*, 1988; Roberts *et al.*, 1988). Currently, variation in gp120 is understood to reflect an evolutionary struggle between the host immune system and the virus, with circulating sequences reflecting viruses not yet recognized by the host immune response. gp120 is characterized as containing five variable regions (V1–V5) sequentially occurring in the primary sequence with five relatively conserved regions (C1–C5) (Starcich *et al.*, 1986; Willey *et al.*, 1986). Sequence analyses also identified 18 highly conserved cysteine residues that form nine disulfide bonds that play an important role in the formation of gp120 tertiary structure (Leonard *et al.*, 1990). The variable regions determined by sequence analyses coincided with loop structures that were predicted to be formed by the disulfide bonds (Leonard *et al.*, 1990). The presence of these loops has been confirmed by X-ray crystal structures of gp120 proteins (Huang *et al.*, 2005; Kwong *et al.*, 1998). In addition, gp120 contains 20–30 canonical *N*-linked glycosylation sites and is extensively glycosylated with oligosaccharides accounting for approximately half of its molecular mass. The location and number of glycosylation sites also varies by strain and change over time during the course of infection (Allan *et al.*, 1985; Montefiori *et al.*, 1988). The variations in sequence and the uniquely dense glycosylation are thought to be important mechanisms by which the virus evades the humoral immune response.

**Fig. 3.**
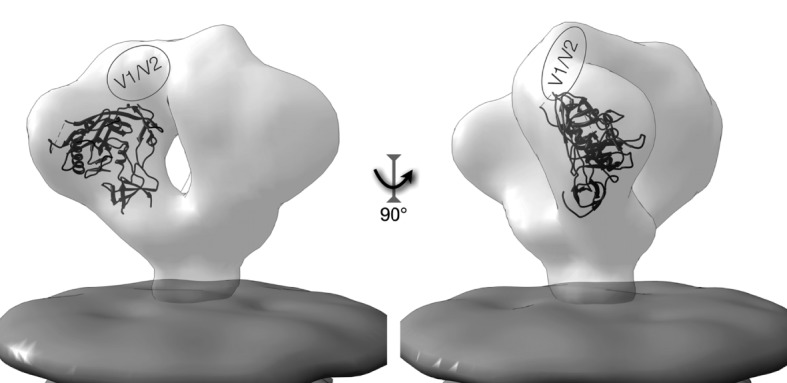
Structure of virion-associated Env. The structure of HIV Env is presented with the relative localization of a gp120 subunit within the virion-associated trimeric structure. The Env volume is displayed in light grey, while the membrane is darker grey at the bottom of the figure. The figure was constructed using the EM Database files EMD-5019 (Env) and EMD-5022 (membrane) (Liu *et al.*, 2008) and the gp120 co-ordinates from PDB file 2NY7 (Zhou *et al.*, 2007), with gp120 positioned into the EM density map using the ‘Fit in Map’ tool in UCSF Chimera (Pettersen *et al.*, 2004).

HIV-1 gp120 has been studied extensively, and a number of atomic resolution structures have been elucidated, both in the unliganded form (Kwon *et al.*, 2012) and in conjunction with one or more binding partners, including CD4 and co-receptor-binding site mAbs 17B (Kwong *et al.*, 1998) and X5 (Huang *et al.*, 2005), or CD4-binding site mAbs b12 (Zhou *et al.*, 2007), VRC01 and VRC03 (Wu *et al.*, 2010). Due to what is likely extensive flexibility, the V1/V2 loops were deleted from gp120 proteins used for structure determination (Kwong *et al.*, 1998). The relative location of the V1/V2 loops on the virion-associated Env trimer has been recently determined, however, by fitting (V1/V2 deleted) gp120-CD4 crystal structures into cryo-electron microscopy (cryo-EM) density maps of CD4-bound Env (Liu *et al.*, 2008). The inclusion of CD4 in the cryo-EM Env structure determination allowed for unambiguous orientation and fitting of pre-determined CD4/gp120 crystal structures that led to the identification of V1/V2 density in the cryo-EM Env structure (Fig. 3) (Liu *et al.*, 2008; White *et al.*, 2010).

## gp41 structure and function

gp41 can be structurally sub-divided into three major domains (see Fig. 2): the extracellular domain (or ectodomain); the membrane-spanning domain (MSD); and the C-terminal tail (CTT), also referred to classically as the intracytoplasmic tail. Of these, experimental protein structures for only the gp41 ectodomain have been determined at atomic detail, and then only for the six-helix bundle of what is generally considered the fusion-competent state (Caffrey *et al.*, 1998; Chan *et al.*, 1997; Shi *et al.*, 2010). Little to no structural information exists on the native structure of gp41. Even recent cryo-EM structures of Env on the virion surface provide little insight into the structure of gp41 beyond that it appears to present as a thin stalk supporting the gp120 domains (Liu *et al.*, 2008; White *et al.*, 2010).

## gp41 ectodomain

The location and function of the gp41 ectodomain is well-studied and generally unquestioned. gp41 is also *N*-glycosylated, although to a much lesser extent than gp120, with three to five potential sites (Montefiori *et al.*, 1988). Initial modelling studies of the gp41 ectodomain from the primary amino acid sequence revealed a remarkable similarity of the predicted structure to the crystal structure of the influenza HA protein, despite the lack of significant amino acid sequence homology (Gallaher *et al.*, 1989). Subsequent structural studies of gp41 ectodomain sequences, including X-ray crystallography, confirmed the predicted structure of the gp41 ectodomain. The ectodomain contains the major determinants of membrane fusion (Fig. 2): a hydrophobic N-terminal domain termed the fusion peptide (FP) (Bosch *et al.*, 1989; Freed *et al.*, 1990, 1992); two heptad repeat regions, termed HR1 and HR2 (also referred to as the N- and C-helix, respectively) that form α-helical coiled-coil structures and are linked by a disulfide-bridged loop (Caffrey *et al.*, 1998; Chan *et al.*, 1997; Dubay *et al.*, 1992; Lu *et al.*, 1995; Weissenhorn *et al.*, 1997); and a tryptophan-rich region referred to as the membrane-proximal external region (MPER) (Muñoz-Barroso *et al.*, 1999; Salzwedel *et al.*, 1999). Additionally, the MPER is an important target for broadly neutralizing mAbs (Montero *et al.*, 2008).

**Fig. 4.**
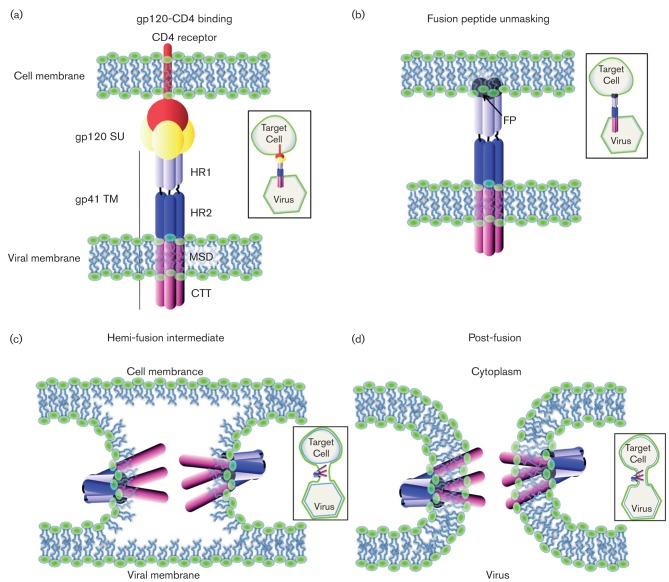
Schematic representation of the virus and cell fusion. (a) Virus binds to the target cell through the interaction of gp120 SU with CD4 cellular receptor. (b) This interaction activates a conformational change of the viral envelope leading to unmasking of the FP from gp41, which inserts in the cellular plasma membrane. The segment portion of HR2 interacts with HR1 in an anti-parallel way in the trimer, forming the six helix bundle. (c) This conformational change of the envelope brings the viral and cellular plasma membrane in close proximity, allowing their fusion. Only the external leaflets of both membranes are fused such that this step is called the hemi-fusion intermediate. (d) Both external and inner leaflets of the membranes are fused, leading to the complete fusion.

The primary function of the gp41 ectodomain is to drive virus–cell membrane fusion. The gp41 FP is normally buried in the gp120/gp41 complex, but following conformational rearrangements induced by receptor and co-receptor binding, FP is exposed and inserts into the target cell membrane. The three HR1 domains then fold into a stable six-helix bundle in an anti-parallel fashion with the three HR2 domains (Fig. 4) to bring the virus and cell into close proximity to allow fusion to occur (Chan *et al.*, 1997; Melikyan, 2011; Weissenhorn *et al.*, 1997). The mechanism by which the actual fusion step occurs is not well elucidated. However, peptide analogues of both the FP and the MPER have been demonstrated to both disorder and reduce the bending modulus for lipid bilayers, and thus may lower the free energy barrier to membrane fusion, a decidedly non-spontaneous process (Greenwood *et al.*, 2008; Tristram-Nagle *et al.*, 2010; Tristram-Nagle & Nagle, 2007).

## gp41 membrane-spanning domain

In contrast to the gp41 ectodomain, the gp41 MSD remains, despite much study, structurally relatively undefined. Early studies identified a hydrophobic 25 aa sequence that acted as a ribosomal stop-transfer signal, leading to the classical identification of the gp41 ectodomain, MSD, and intracytoplasmic tail (Haffar *et al.*, 1988). The MSD amino acid sequence is highly conserved among HIV isolates, and contains a conserved GXXXG helix–helix interaction motif and a highly conserved midspan arginine residue. The classical structural view of the MSD is that of a single membrane-spanning α-helix. The presence of the midspan arginine, as well as results from both previous and more recent studies, however, lead to conflicting views of the sequence and structural characteristics of the MSD in contrast to the classical definition. These studies are briefly discussed below.

**Fig. 5.**
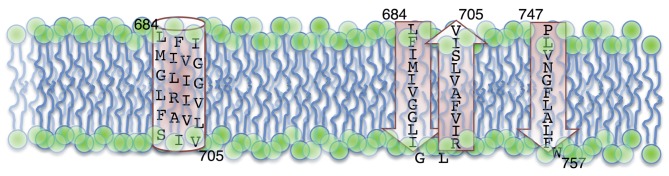
Proposed models for the membrane-spanning sequences of HIV gp41. The two models for the HIV gp41 MSD are shown schematically using the sequence from HIV-1 89.6. (Left) The traditional model, with the MSD as a single α-helix. (Right) The alternative model proposed by Hollier & Dimmock (2005) with three membrane-spanning β-sheets. Residues are numbered according to the standard HXB2 sequence, and residues 706–746 are left out for simplicity.

Prior studies attempting to generate antibodies (in rabbits) to peptides spanning the full Env protein (gp120 and gp41) identified a strongly reactive peptide derived from positions 728 to 745 that is referred to as the Kennedy epitope (KE) (Chanh *et al.*, 1986; Kennedy *et al.*, 1986) located in the CTT. Intriguingly, the sera directed to the KE strongly neutralized virus (Chanh *et al.*, 1986), suggesting an external localization of the epitope on the virion, as antibodies cannot cross intact lipid membranes. These results were largely dismissed in light of the identification of the stop-transfer signal and the division of gp41 into the classical external, membrane-spanning and intracytoplasmic domains (Haffar *et al.*, 1988). More recently, however, accumulating evidence also points to possibly different MSD sequence and/or structure. Studies examining the exposure of the KE on the surface of Env-expressing cells suggest that under some circumstances the KE is extracellularly exposed, indicating a need for either additional MSD sequences, or an alternative structure for the MSD other than the traditionally held α-helix (Cheung *et al.*, 2005; Cleveland *et al.*, 2003; Heap *et al.*, 2005; Reading *et al.*, 2003). The two proposed MSD models are shown in Fig. 5.

One of the most successful techniques for examining the structure of the MSD has been the use of molecular dynamics (MD) simulations. Two recent studies use MD simulations to examine the stability of monomeric and trimeric forms of the MSD in lipid membranes (Kim *et al.*, 2009) and conformational variation in the MSD (Gangupomu & Abrams, 2010). A general caveat of these techniques, however, is that the starting assumptions can have a major impact on the outcome. As such, both studies begin with the assumption that the MSD sequence adopts a helical conformation in the membrane. In general, this is a reasonable assumption given that the most energetically favourable conformation of a peptide in a membrane is an α-helix due to the internal stabilization of all potential hydrogen-bonding partners (White & Wimley, 1999). It does not, however, account for the experimental data that suggests alternative gp41 topologies, and thus alternative sequences or structures for the MSD. With that understanding, both studies provide interesting insights into the potential structure of the MSD. Engelman and colleagues demonstrated a remarkable stability of the HIV MSD sequence (using FIMIVGGLVGLRIVFAVLSI) as a monomeric helix in the lipid membrane, but rapid unfolding of the helix in water (Kim *et al.*, 2009). They also demonstrated conformational stability of a right-handed helical bundle that increased further when the central arginine residues were deprotonated (Kim *et al.*, 2009). These results suggest that a right-handed helical bundle with deprotonated arginine residues is the most stable trimeric conformation for the gp41 MSD sequence. In contrast, Gangupomu and Abrams examined the conformational flexibility of a monomeric MSD sequence (using KLFIMIVGGLVGLRIVFAVLSIVNRVR). Their findings indicate that the MSD monomer forms a stable-tilted helix in the membrane, but that metastable states are formed when the midspan arginine ‘snorkels’ to either side of the membrane (Gangupomu & Abrams, 2010). When the arginine snorkels towards the outer leaflet, the N-terminal end of the MSD is unfolded. However, when the midspan arginine snorkels towards the inner leaflet the MSD kinks at phenylalanine 697, and the remaining 10 aa form a helix along the inner leaflet at the water–membrane interface (Gangupomu & Abrams, 2010). Collectively, and importantly, these MD simulation results suggest multiple (meta)stable conformations of the MSD, even in an α-helical state.

Functionally, the primary role of the MSD is to anchor the Env protein to the cellular and viral membrane. Additional studies have identified a role for the MSD in overall Env function. In particular, mutations in the MSD have been shown to have an effect on Env-mediated fusion such that mutations of conserved residues in the MSD, including the midspan arginine, led to decreased cell–cell fusion and decreased viral infection (Kondo *et al.*, 2010; Shang & Hunter, 2010; Shang *et al.*, 2008; Yue *et al.*, 2009). The mechanisms by which these mutations act to decrease fusion are not well understood.

## gp41 C-terminal tail

### Structure

Of the three gp41 domains, the structure of the gp41 CTT is the least understood. Results from early topogenesis studies led to the view of gp41 (and thus Env as a whole) as a type I membrane protein, with an extracellular N terminus, a single MSD, and an approximately 150 aa long cytoplasmic CTT (Haffar *et al.*, 1988). Comparisons of this model to other retroviral Env proteins supported this view, with a major difference in the length of the proposed cytoplasmic CTT. For most retroviral Env proteins, the cytoplasmic CTT sequences are generally between 20 and 40 aa long (Checkley *et al.*, 2011). The presence of a very long CTT is not unique to HIV, as other lentiviruses such as the simian immunodeficiency virus (SIV) and the equine infectious anemia virus have similarly long CTTs, at 150 and 200 aa, respectively (Rushlow *et al.*, 1986). The presence of a long CTT in most lentiviruses suggests an important functional role, as viruses do not generally replicate non-functional sequences. This functional importance is supported by the finding that truncation of the CTT leads to *in vivo* suppression of viral replication in animal models (Shacklett *et al.*, 2000). A decade earlier, however, studies demonstrated that the CTT was dispensable for *in vitro* viral replication (Chakrabarti *et al.*, 1989; Hirsch *et al.*, 1989; Kodama *et al.*, 1989), leading to a long-held view that the CTT was not functionally important. This view was subsequently popularized by the finding that truncation of the CTT led to increased Env incorporation into the virion, which was an important consideration at the time as a means by which to boost the anti-Env immune response in vaccine studies. It is now generally accepted that the CTT plays multiple important functional roles in the virus life cycle. Structurally, very little is still known and no atomic level structures exist for full-length CTT sequences.

Structural information has been determined for some CTT sequences. The most well-studied domains, the lentivirus lytic peptides (LLPs), are sequences that were initially identified by sequence scanning as having extraordinarily high hydrophobic moments (Eisenberg & Wesson, 1990; Miller *et al., 1991*). Subsequent studies on peptide analogues of these domains demonstrated high levels of structural similarity with naturally occurring cytolytic amphipathic cationic anti-microbial peptides (Miller *et al.*, 1991) as well as the ability of the peptides to alter cell membrane permeability (Miller *et al.*, 1993a). Peptide analogues of these domains have been demonstrated to be generally unstructured in aqueous buffer, but to rapidly adopt amphipathic α-helical structure in membrane or membrane-mimetic environments as determined by circular dichroism spectroscopy (Chernomordik *et al.*, 1994; Kliger & Shai, 1997; Srinivas *et al.*, 1992; Steckbeck *et al.*, 2011). There are, however, no atomic level structures of the peptides interacting with membranes or membrane mimetics. Recently, the physico-chemical and structural characteristics of the LLPs were determined to be highly conserved among sequences from numerous clades and groups, despite substantial variations in primary amino acid sequences (Steckbeck *et al.*, 2011). In particular, conservation of the membrane-associating amphipathic α-helical structures suggests that the LLP regions may function in anchoring the CTT to the cellular and viral membranes. In addition, the higher and unusual degree of conservation of arginines in the LLP sequences might be of importance in regulating the interaction of the CTT with the membrane (Steckbeck *et al.*, 2011).

## Topology and function

As the intracytoplasmic localization of the CTT has been predominantly reinforced implicitly with functional data, the topology and function of the CTT will be presented together. As discussed above, the topology of the CTT has long been considered to have an entirely cytoplasmic localization. Accumulating recent evidence suggests that this may not be the case, or is at least not always the case, and will be developed further in the next section. Early topogenesis studies suggested the organization of Env as a type I membrane protein (Haffar *et al.*, 1988), a view that is predominant and has been implicitly reinforced by a number of experimental functional studies. This is largely due to genetic evidence demonstrating functional interactions of the CTT with known intracellular or intravirion partners. These functional interactions have been found to regulate, or be regulated by: (i) Env incorporation into viral particles (Freed & Martin, 1995; 1996; Jiang & Aiken, 2007; Murakami, 2008; Murakami & Freed, 2000a, b); (ii) virion maturation (Jiang & Aiken, 2007; Joyner *et al.*, 2011; Kol *et al.*, 2007; Wyma *et al.*, 2004); and (iii) endocytosis of Env from the cell surface to late endosomes (Byland *et al.*, 2007; Ohno *et al.*, 1997).

## Virion Env incorporation

In the mid-1990s, studies were published that demonstrated a functionally important role for the CTT. These studies are crucial to the current CTT field in that they established conclusively that the CTT was of functional importance, in contrast to the view that arose from studies published in the late 1980s, indicating that the CTT was dispensable for virus replication (Chakrabarti *et al.*, 1989; Hirsch *et al.*, 1989; Kodama *et al.*, 1989). In addition to the effect of CTT-deletion on replication, these studies demonstrated increased Env content in virions containing a CTT-deleted Env and proposed that deletion of the CTT would be a means to boost the anti-Env immune response through the increase in Env per virion. This was the prevailing attitude until seminal studies by Freed and Martin demonstrated that the CTT was implicated in the incorporation of Env into virions through interactions with the MA domain of Gag. Their first study used site-directed mutagenesis to demonstrate that mutations in specific amino acids in Gag MA resulted in deficiencies in incorporation of Env with a full-length CTT (Freed & Martin, 1995). The MA mutations did not, however, lead to reduced incorporation of heterologous retroviral Env proteins with naturally short CTT sequences. Further, specific truncation of CTT sequences to seven or 47 residues (from the original 150) reversed the Env-incorporation block imposed by the MA mutations. This paper has demonstrated for the first time conclusive evidence of a functional role for the CTT through direct or indirect interactions with an intracellular partner, Gag MA.

The role and localization of sequences important in the MA–CTT interaction has been further elucidated. A subsequent paper by the same authors demonstrated that truncating up to 56 residues from the CTT (leaving a 94 residue CTT) eliminated Env incorporation in viruses containing MA mutations leading to deficiencies in Env incorporation, but that truncations of 93 aa or greater (leaving only 57 aa CTT) relieved the block caused by MA mutations, resulting in efficient Env incorporation (Freed & Martin, 1996). Later papers from this group localized the MA–CTT interaction to LLP2 in the CTT (Murakami & Freed, 2000a) and further demonstrated that the interaction was crucial for Env virion incorporation in a cell-type-specific manner (Murakami & Freed, 2000b). It remains unclear whether the interaction between MA and the CTT occurs through a direct or indirect mechanism. In 1996 Cosson published the first, and to date only, *in vitro* demonstration of a direct interaction between these two proteins by GST pulldown (Cosson, 1996). However, in 2006 another group showed that a cellular protein, TIP47 (tail-interacting protein of 47 kDa) was necessary for a functional interaction between Env and Gag (Lopez-Vergès *et al.*, 2006). The role of TIP47 in the Env–Gag interaction is currently unclear, and further studies will be necessary to determine whether the Gag–Env interaction occurs through a direct or indirect mechanism (Checkley *et al.*, 2011).

The Env–Gag interaction is also of importance for the correct localization of Env in the plasma membrane and consequently for the efficient incorporation of Env in the virions. Indeed, the processed Env protein is transported to the plasma membrane and more specifically to the lipid rafts where it interacts with Gag, participating in viral assembly and virus budding (Nguyen & Hildreth, 2000). The palmitoylation of two cysteines in the cytoplasmic domain of gp41 has been indicated as necessary for the correct targeting of Env to these regions (Bhattacharya *et al.*, 2004; Rousso *et al.*, 2000). Later, it was shown that Gag was responsible for the recruitment of Env to the lipid rafts (Bhattacharya *et al.*, 2006; Patil *et al.*, 2010) and that palmitoylation of cysteines was not involved (Chan *et al.*, 2005). Indeed, deletion or mutation of Gag renders Env unable to localize to lipid rafts, limiting its incorporation into virions (Bhattacharya *et al.*, 2006; Patil *et al.*, 2010). Interaction with Gag and a membrane-proximal tyrosine-based motif overlapping the endocytosis motif in the CTT have also been shown to be important to polarized assembly and release of the virus (Deschambeault *et al.*, 1999; Lodge *et al.*, 1994, 1997). Whether the interaction between Gag and Env occurs at the plasma membrane or during their transport to the plasma membrane has not yet been determined.

Some published data suggest a role for the interaction between Env and Gag in HIV infectivity during virion maturation, as described below.

## Virion maturation

Aiken and colleagues examined virion organization by characterizing the effect of pelleting immature HIV virus particles (in which the Pr55^Gag^ has not been cleaved) through detergent (Wyma *et al.*, 2000). Pelleting particles through detergent strips away the viral lipid membrane in which the CTT is embedded, leaving viral protein cores. Their reasoning was if the CTT and Pr55^Gag^ interact, then gp41 should be associated with the pelleted viral cores. They found that the pelleted cores retained the major fraction of the gp41 found in untreated virions (Wyma *et al.*, 2000). Virions treated similarly but with a truncated CTT did not retain the gp41 with the pelleted cores (Wyma *et al.*, 2000). This study does not preclude, however, the presence of a mediating protein that facilitates the interaction between gp41 CTT and PR55^Gag^. Interestingly, a previous study using mature viral particles for a similar analysis demonstrated no association of the CTT with MA (Kotov *et al.*, 1999). Thus, taken together, these results suggested that the association of the CTT with Pr55^Gag^ is dependent on the maturation state of the virion.

This maturation-dependent association of the CTT with Gag has been extended to examine the role of the CTT in viral fusion. It is well established that HIV particles are not infectious until proteolytic cleavage of the Pr55^Gag^ into its constituent domains (MA, CA and NC, predominantly). Using a reporter assay to measure virus–cell fusion, Wyma *et al.* demonstrated that immature virions were less fusogenic than mature virions in a manner that was dependent on the CTT, as truncations of the CTT resulted in identical fusogenicity of immature and mature viral particles (Wyma *et al.*, 2004). More specifically, the interaction of gp41 CTT with an unprocessed Gag inhibits fusion, which is restored by Gag processing or CTT deletion (Murakami *et al.*, 2004). More recent results suggest that the extreme C terminus of the CTT, LLP1, modulates the maturation dependence of infectivity, while deletion of this region does not affect the incorporation of Env into immature viral particles (Jiang & Aiken, 2007). As such, the interaction between the CTT and Gag is regulated by the maturation state of the viral particles, thereby modulating fusion and thus the efficiency of infectivity. The CTT has also been shown to modulate the mechanical stability of immature virus particles relative to mature virus particles, presumably through its interactions with Pr55^Gag^ (Kol *et al.*, 2007). These results collectively demonstrate that the interaction of the CTT and Gag have specific effects on the infectivity and structural stability of HIV particles.

## Env endocytosis

Another functional role of the CTT has implicitly reinforced the traditional topological model. The gp41 CTT of both SIV and HIV have long been known to contain endocytic signals. This was first demonstrated in HIV-1 in a study of the Env internalization pathway that allows processing for antigen presentation by the class II MHC (Rowell *et al.*, 1995). In this study the authors observed a high rate of Env internalization that was dependent on a tyrosine residue located six positions from the MSD. In parallel, similar observations were reported for SIV where the CTT had been shown to contain an endocytic signal (Sauter *et al.*, 1996). The transfer of the SIV CTT to a chimeric CD4 fusion protein (external CD4 domain associated to the MSD and CTT of SIV) resulted in the internalization of the chimeric protein in a manner dependent on ^703^tyrosine (Sauter *et al.*, 1996). A consensus YXXΦ motif in the CTT of HIV-1 was then shown to interact with members of the adaptor protein medium chain family (Ohno *et al.*, 1997). It has also been demonstrated that CTT sequences interact specifically with the AP-2 clathrin adaptor (Boge *et al.*, 1998). Most recently, Byland *et al.* demonstrated that the CTT contains two functional endocytic signals: a ^711^GYXXΦ motif located near the N terminus of the CTT, and a dileucine motif at the extreme C terminus (Byland *et al.*, 2007). Their results indicate that in order to completely abolish endocytosis of Env from the cell surface to late endosomes, both motifs must be mutated, suggesting that both the N- and C-termini of the CTT are cytoplasmically localized.

Additional tyrosine and dileucine motifs have been identified in the CTT and studied in a recent publication by Hunter and colleagues, demonstrating that these motifs do not affect cell surface expression of Env, but rather its incorporation into virions, with cell–cell fusion and virus infectivity also being affected (Bhakta *et al.*, 2011), suggesting that these additional motifs are not involved in Env endocytosis. As noted by the authors, a number of these motifs overlap the LLP regions, and the observed phenotypic impairment could be due to alterations of LLP functions. However, the ^802^YW^803^ motif plays a role in internalization through an interaction with TIP47, allowing the Env retrograde transport (Blot *et al.*, 2003). As mentioned previously, TIP47 is thought to bridge Gag and Env and is necessary for Env incorporation into virions (Lopez-Vergès *et al.*, 2006). Thus, the addition of Gag might allow a redirection of Env from an internalization process to incorporation into virions (Lopez-Vergès *et al.*, 2006).

**Fig. 6.**
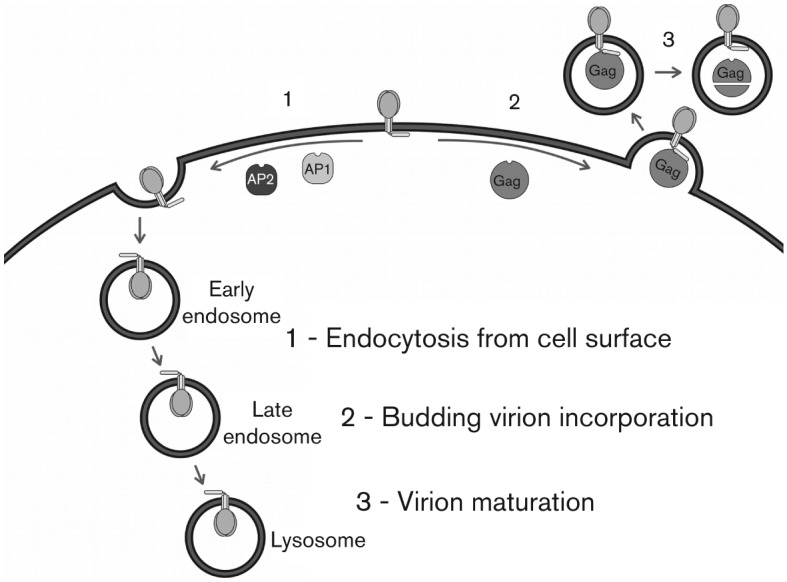
CTT function. Known functional roles of the CTT are presented schematically. (1) The CTT has been demonstrated to influence endocytosis of Env from the cell surface through interactions with AP1 and AP2. (2) Through interactions with Gag, the CTT plays a role in incorporation of Env into budding virions. (3) The interaction between Gag and the CTT may play a role in virion maturation.

Overall, known CTT functions in Env virion incorporation and maturation as well as endocytosis of Env from the cell surface provide support for the traditional intracytoplasmic (intravirion) model for the CTT. Fig. 6 presents an overview of the known functionalities associated with the CTT sequences.

## CTT cell functions

Little is known about the functions of the CTT beyond its role in trafficking and Env incorporation. Indeed, in addition to the interactions detailed above, the CTT may also have a role during virus entry and in circumventing some cellular functions.

One of the first identified functions of the CTT concerned the LLP domains having a cytolytic and cell–cell fusion inhibition activity as peptides based on functional interactions with lipid membranes (Srinivas *et al.*, 1992). The gp41 subunit also physically interacts with cellular proteins. Indeed, the calmodulin cellular protein binds to the CTT through the LLP1 domain, leading to the inhibition of the calmodulin activity (Miller *et al.*, 1993b; Tencza *et al.*, 1995). Point mutations of arginines in the LLP1 domain are associated with a decreased binding of LLP1 peptides to calmodulin and lead to an impairment of the cytolytic activity (Miller *et al.*, 1993b; Tencza *et al.*, 1995). Interestingly, the LLP peptides also inhibit T-cell activation, one of the calmodulin cellular functions (Srinivas *et al.*, 1993), and Env is associated with an increased sensitivity of T-cells to FAS-mediated apoptosis in an LLP1-calmodulin-binding-dependent manner (Micoli *et al.*, 2000). Thus, these functions of the LLP domains have been proposed to contribute to the cytopathic effect of HIV-1 on T-cells.

Recent studies have demonstrated that the CTT could interact with or affect other cellular pathways. For instance, the HIV-1 CTT has been shown to activate the canonical NF-κB pathway through a direct or indirect interaction with TAK1 cellular protein (Postler & Desrosiers, 2012). This activation appears to be of critical importance in suboptimally activated T-cells. Additionally, the CTT, and more specifically the three LLP domains, has been reported to directly interact with the cellular protein p115-RhoGEF, which is involved in cell proliferation (Zhang *et al.*, 1999). Such an interaction prevented the activation of serum response factor transcription factor, but did not prevent its activation by other factors. In addition, the impairment of this interaction led to a defect in HIV-1 replication in T-cells.

Finally, Luman, a cellular protein belonging to the CREB/ATF family of bZIP transcription factors, also interacts with the CTT (Blot *et al.*, 2006). Luman is anchored in the ER, and following proteolytic cleavage, the truncated, active form of Luman is translocated in the nucleus where it inhibits HIV-1 LTR transactivation by interacting with the viral Tat protein. However, the CTT of gp41 interacts with the ER-embedded inactive form of Luman and decreases the stability of the cellular protein. As such, by acting on the precursor of Luman, gp41 may decrease the amount of active translocated Luman available to inhibit the LTR transactivation by Tat (Blot *et al.*, 2006).

These functions of the CTT are likely of importance for the virus life cycle and disease progression, but significant progress remains to be made to better understand their implications.

## Evidence for alternative CTT topologies

### Surface exposure of the KE

The derivation of an alternative topological model for CTT comes from a combination of indirect (inferred from neutralization of HIV infection by anti-CTT antibodies) and direct biochemical evidence. Initial studies suggestive of a non-cytoplasmic localization for the CTT were not explicitly performed to determine the CTT topology, but provided the earliest indirect evidence for potential exposure. These studies found that serum from a rabbit immunized with a gp41 peptide (residues 728–745, the KE) bound to HIV Env, and that serum from HIV-1-infected patients bound the synthetic peptide (Kennedy *et al.*, 1986). Subsequent studies demonstrated that anti-KE serum could specifically neutralize HIV *in vitro* (Chanh *et al.*, 1986). As antibodies cannot cross intact lipid membranes, these results implied the localization of the KE on the outer surface of the virion, in direct contrast to the presumed intravirion localization.

**Fig. 7.**
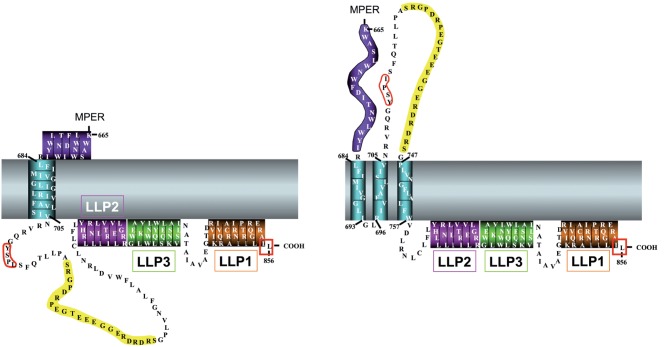
Proposed topology models for HIV gp41 CTT. The two predominant CTT topology models are presented. (Left) Traditional CTT topology model, with completely intracytoplasmic CTT. (Right) model proposed by Hollier & Dimmock (2005), with the KE (yellow) externally localized. Transmembrane domains are as presented in detail in Fig. 5. Red boxes indicate functional endocytic sequences.

More recently, the exposure of CTT sequences has been explored predominantly by Nigel Dimmock’s lab (for review see Dimmock, 2005). Over 3 years and five manuscripts, his group provided evidence for the extravirion and/or extracellular localization of CTT sequences, and it is his group that proposed the alternative topological model that is contrasted with the traditional model in Fig. 7.

The initial evidence for external localization of the KE sequences were the result of antibody neutralization and binding studies of anti-CTT antibodies to viral particles (Cleveland *et al.*, 2003). This study used epitope-purified antiserum (against the epitope ^746^ERDRD^750^ and termed EPES) to demonstrate high levels of neutralization [concentration necessary to achieve 50 % neutralization (NC_50_) = 0.3 µg ml^−1^] of HIV *in vitro*. Importantly, EPES did not have any neutralizing activity against viruses with a CTT-deleted Env. They further demonstrated that EPES could bind to intact viral particles (where intact particles were demonstrated by a lack of binding by anti-MA and anti-CA antibodies), and that this binding was abolished by protease treatment of the particles (Cleveland *et al.*, 2003). This report provided the first direct evidence for exposure of CTT sequences on viral particles.

A second study from Dimmock’s group described a mAb, termed SAR1, that is directed against the KE, as well as its neutralization activity (Reading *et al.*, 2003). SAR1 was found to bind to both soluble peptides and proteins containing a GERDRDR sequence. More interesting, however, was that SAR1 bound to cells infected with all different HIV strains tested, but only to some virions representing the same strains. Moreover, SAR1 did not exhibit neutralization activity in a standard neutralization assay, but was able to neutralize effectively in a post-attachment neutralization (PAN) assay. In a standard neutralization assay, antibody and virus are incubated together prior to adding to the cells to allow the interaction to reach equilibrium before addition to target cells; neutralizing antibodies reduce viral infection in a concentration-dependent manner. In a PAN assay, however, antibody, virus and target cells are incubated together, but at temperatures that do not support virus–cell fusion (usually ≤31 °C) for a defined period of time before washing free virus from the cells and incubating at 37 °C to allow infection to occur (or not, if the antibody neutralizes) (Reading *et al.*, 2003). The inability of SAR1 to neutralize in a traditional assay, but exhibit PAN activity, suggested a transient exposure of the KE during the fusion process. The mechanism of the PAN activity of SAR1 was confirmed in a subsequent paper to act through the blocking of virus–cell fusion (Heap *et al.*, 2005). Reading *et al.* also demonstrated that SAR1 acted to block the production of infectious progeny virus from infected cells (Reading *et al.*, 2003). These results provided increased evidence for an alternative topology for the CTT, where the KE is exposed to antibody binding, although not universally as SAR1 did not bind to virions from all HIV strains tested. Indeed, it is noteworthy that the KE seems to be exposed differently at the surface of virions and of the cells. Recent studies have demonstrated that two different mAbs directed against the KE (Chessie 8 and SAR1) bind exclusively at the surface of the cells and not to virions, suggesting different conformations for the CTT that are dependent on context (Cheung *et al.*, 2005; Steckbeck *et al.*, 2010). However, the cell surface exposure of the CTT in SIV has recently been proposed to be a consequence of Env shed from transfected cells binding non-specifically to other cells in culture thus exposing the CTT to antibody binding (Postler *et al.*, 2012). A conclusive model for functional CTT topology remains to be elucidated.

Finally, in an effort to provide a condensation of results from these studies and to provide a working topological model explaining their results, Hollier and Dimmock used sequence analyses to provide theoretical support to their observed exposure of CTT sequences. Using a combination of secondary structure predictions with hydrophobicity analyses, Hollier and Dimmock proposed that the CTT might form a tail loop structure supported by three membrane-spanning β-sheets that result in the extracellular localization of the KE (Hollier & Dimmock, 2005). This is the alternative topological model presented in Fig. 7. Hollier and Dimmock acknowledge that their proposed topological model positions the N-terminal GYXXΦ endocytic signal in an extracellular position, implying that it cannot be functional. As discussed above, this model is in direct contrast with subsequent results, demonstrating the GYXXΦ signal to be active in AP-2-mediated endocytosis (Byland *et al.*, 2007). Hollier and Dimmock propose that the majority of cell surface Env is in a state similar to the traditional topological model, with a single MSD, but that a minor population exists in their newly proposed topological state, and that it is this KE-exposed Env that is preferentially incorporated into progeny virions (Hollier & Dimmock, 2005). A major argument against this model is the improbable existence of an MSD composed entirely of β-sheet with an odd number of membrane-interacting sequences. One of the ‘construction rules’ of β-sheet membrane proteins is that they must have an even number of MSDs, as β-sheets hydrogen bond most efficiently in an anti-parallel fashion (Schulz, 2000). An odd number of β-sheet MSD sequences would leave a lone β-strand and its hydrogen-bonding backbone amide and carboxyl groups exposed to the hydrocarbon interior of the membrane, an energetically unfavourable situation (Schulz, 2000). This unfavourable arrangement would be further compounded upon Env trimerization, where there would now be three naked β-sheets in the interior of the membrane, all arranged in a parallel fashion and unable to relieve the unfavourable interactions.

In contrast to the Hollier and Dimmock model, other recent studies support the incorporation of a single MSD gp41 in virions. It has been demonstrated that HIV-1 develops a resistance to antibiotic amphotericin B methyl ester through mutations in gp41 that create proteolytic cleavage sites in the cytoplasmic tail that allow truncation of the CTT by the viral protease in the virions, but not in cells (Waheed *et al.*, 2007; Waheed *et al.*, 2010). This observation is consistent with the existence of different Env conformations at the virus and cell surfaces which could be responsible for different accessibility of the cleavage sites to the protease (Waheed *et al.*, 2007). Interestingly, 10 % of gp41 molecules are not cleaved in virions, suggesting the possible co-existence of alternative CTT structures as a minority population (Waheed *et al.*, 2007). By contrast, these CTT cleavage sites are inaccessible in cells, suggesting that either interaction with viral or cellular proteins or an alternative CTT conformation prevents protease access and cleavage.

## Dynamic exposure of LLP2 sequences

In addition to the evidence for possible KE exposure, recent studies present data supporting the exposure of additional CTT sequences, in particular the LLP2 domain. A study by Lu *et al.* determined the exposure of CTT sequences in Env-expressing cells under native conditions and during the fusion process (Lu *et al.*, 2008). Using antibodies directed at the C-terminal 90 aa of the CTT and to the LLP2 region only, Lu *et al.* determined that no exposure of the C-terminal 90 aa was detected under normal conditions, nor did they observe exposure during cell–cell fusion carried out at 37 °C as measured by flow cytometry. They did, however, observe LLP2 exposure during cell–cell fusion by slowing the fusion reaction by incubation at 31.5 °C (Lu *et al.*, 2008). These same antibodies were also demonstrated to inhibit cell–cell fusion in a concentration-dependent manner at 31.5 °C, but not at 37 °C. Their results suggest a transient exposure of LLP2 sequences during membrane fusion, and are consistent with results demonstrating membrane association of LLP2 both pre- and post-fusion (Viard *et al.*, 2008).

## CTT influences overall Env structure

The final known function of the CTT is its role in modulating overall Env structure and functional properties. One of the earliest observations that the CTT could influence overall Env structure was done in SIV and showed that truncation of the CTT increased the accessibility of the reactive amino acids from the envelope in a cell surface biotinylation assay (Spies *et al.*, 1994). Further evidence of this phenomenon was provided by the insight that HIV-1 with CTT-deleted Env proteins could infect target cells in a CD4-independent manner (Edinger *et al.*, 1997). Until that time, the paradigm for HIV infection of target cells was that gp120 binding to CD4 was necessary to induce conformational changes that allowed binding to co-receptor. Edinger *et al.* demonstrated that viruses with a CTT-deleted Env were able to infect CD4 negative/co-receptor-positive cells, suggesting the existence of different conformations of Env that are dependent on the presence or absence of the CTT.

Initially, direct evidence for CTT-dependent alterations in Env structure was provided by differential reactivity between CTT-deleted and wild-type Env with conformationally dependent antibodies. Edwards *et al.* demonstrated that truncation of the CTT to 27 aa resulted in increased binding by mAbs directed to both the CD4-binding site and the CD4-induced (CD4i) co-receptor-binding site (Edwards *et al.*, 2002). In Env with a full-length CTT, mAbs directed to the co-receptor-binding site only bound if the Env was preincubated with soluble CD4. The study also demonstrated differential reactivity of conformational mAbs directed to the ectodomain of gp41 between CTT-truncated and full-length CTT (Edwards *et al.*, 2002). This study was the first to demonstrate that the CTT modulates the conformation of overall Env structure, even in the non-covalently attached gp120 that is presumably on the opposite side of the membrane. These results were further confirmed by a study from Wyss *et al.* showing that the CTT truncation not only exposed the CD4i epitope, but also increased fusion efficiency (Wyss *et al.*, 2005). Subramaniam and colleagues recently published cryo-EM structures of CTT-deleted Env on the surface of SIV viral particles (CP-mac variant) (White *et al.*, 2010). In comparison to wild-type virus containing the full-length CTT, CTT-deleted Env existed in a naturally ‘open’ state, displaying large differences in the localization of electron density that are consistent with the conformational changes normally associated with CD4 binding. The CP-mac variant also contains mutations in the gp120 and gp41 ectodomains that may contribute to the open conformation (LaBranche *et al.*, 1994), as other studies have demonstrated that deletion of the CTT alone does not confer a CD4-independent phenotype in HIV-1 (Edwards *et al.*, 2001). Indeed, the combination of CTT truncation and gp120 mutations allows the CD4 independence, indicating that the CTT deletion is necessary but not sufficient to confer a CD4-independent phenotype (Edwards *et al.*, 2001). Other studies, discussed below, provide additional evidence for the influence of the CTT on overall Env structure.

Kalia *et al.* also demonstrated that alterations in the CTT could modulate the antigenic conformation of both the gp120 and gp41 ectodomains. Instead of utilizing large deletions in the CTT, however, Kalia demonstrated that mutation of two conserved arginine residues in LLP2 to glutamate was sufficient to alter the conformation of both gp120 and the gp41 ectodomain on the surface of Env-expressing cells (Kalia *et al.*, 2005). The mutations were shown previously to have no effect on the levels of virion Env incorporation or viral replication, but did decrease the efficiency of cell–cell fusion (Kalia *et al.*, 2003). In addition to demonstrating antigenic distinctions between wild-type and mutant Env on the cell surface, differences were seen in the viral sensitivity to antibody-mediated neutralization by antibodies directed at the CD4-binding site, with the mutant virus demonstrating an approximately 40-fold decrease in neutralization sensitivity (Kalia *et al.*, 2005). This study importantly demonstrated that point mutations in the CTT were sufficient to alter overall Env conformation and antigenicity, and additionally provided the first data regarding the critical nature of the conserved arginine residues in the LLP regions. In addition, this study revealed the potential role of minor CTT variation in Env antigenic variation and immune escape.

A recent paper has extended the study of CTT-dependent alterations in overall Env antigenicity from the cell surface to the surface of viral particles (Joyner *et al.*, 2011). While investigating maturation-induced cloaking of neutralization epitopes, Joyner *et al.* demonstrated that Env on immature virions (protease deleted) reacted differently with a number of conformationally dependent mAbs in a manner that was dependent on the presence or absence of the CTT (Joyner *et al.*, 2011). This study provided the first direct evidence that the CTT plays a major role in modulating the conformation of the CTT in the virion in addition to being on the cell surface.

The potential exposure of the CTT and its modulation of Env conformation could also have some implications in the virus life cycle. Indeed, it has been shown in T-cells that the virus neutralizing EPES antibody binding to the KE was able to inhibit the cell–cell fusion between an HIV-1-infected cell and its target uninfected cell, suggesting a role for the CTT exposure at the cell surface in the virus propagation (Cheung *et al.*, 2005). These results suggest that the presence of the CTT may be important to a dynamic change of conformation of the envelope during the cell–cell fusion. The binding of this antibody to KE epitope could prevent the conformational modification of the envelope at the cell surface and thus inhibit cell–cell fusion. A summary of the different antibodies binding to the CTT and their characteristics is presented in Table 1.

**Table 1.** List of the published antibodies binding to the gp41 CTT na, Not available.

Name of the antibody	Targeted region	Epitope sequence	Epitope characteristics	Species/clonality	Antibody characteristics	References
Chessie 8	KE	PDRPEG	Conformational	Murine IgG	Non-neutralizing binds to HIV-infected cells	Abacioglu *et al.* (1994)
SAR1	KE	GERDRDR	Conformational	Murine IgG	PAN-neutralizing binds to HIV-infected cells and most virions, no binding to intact NL4.3 virions	Reading *et al.* (2003), Heap *et al.* (2005), Steckbeck *et al.* (2010)
EPES (epitope-purified ERDRD specific)	KE	ERDRD	Conformational	Murine polyclonal	Neutralizing binds to HIV-infected cells and virions	Buratti *et al.* (1998), Cleveland *et al.* (2000b, 2003)
anti-LLP2	LLP2	na	na	Rabbit polyclonal IgG	na	Lu *et al.* (2008)
1575	KE	IEEE	Linear immunodominant	Murine monoclonal	Non-neutralizing binds to HIV-infected cells and virions	Buratti *et al.* (1996, 1997, 1998), Cleveland *et al.* (2000a), Evans *et al.* (1989), Heap *et al.* (2005), Vella *et al.* (1993)
1577	KE	ERDRD	Conformational	Murine monoclonal IgG	Non-neutralizing binding to HIV-infected cells and virions (Dimmock), no binding to intact virions (Steckbeck), epitope binding inhibited by mAb1575	Beaumont *et al.* (2000), Buratti *et al.* (1996, 1998), Chomont *et al.* (2008), Cleveland *et al.* (2000a, 2003), Evans *et al.* (1989), Holl *et al.* (2006), Steckbeck *et al.* (2010), Vella *et al.* (1993)
1583	KE	ERDRD	Conformational	Murine monoclonal IgG	Non-neutralizing binds to virions	Evans *et al.* (1989), Buratti *et al.* (1996, 1998), Vella *et al.* (1993), Cleveland *et al.* (2003), Heap *et al.* (2005)

## Conclusion and perspectives

Most studies have focused on the gp120 subunit toward developing an HIV-1 vaccine, removing all or much of gp41 (and always the CTT), to increase protein expression and produce a soluble immunogen. However, as has been summarized above, the CTT plays an important role in Env structure and consequently in the virus life cycle, underscoring the importance of using the entirety of Env, including the CTT, in further immunization studies. It may, therefore, be useful to theorize on the means by which the CTT can exert influence on the structure of the Env ectodomain in an effort to provide a framework for understanding the mechanism(s) by which the influence occurs.

The conservation of the physico-chemical properties is particularly interesting for the LLP regions in light of their proposed membrane associating characteristics (Chernomordik *et al.*, 1994; Fujii *et al.*, 1992; Kliger & Shai, 1997; Srinivas *et al.*, 1992), where hydrophobic moment and charge may complement one another. The hydrophobic moment has been proposed as a measure of the tendency of a sequence to prefer the chemically complex interfacial boundary between the hydrocarbon membrane interior and the aqueous phase (Eisenberg *et al.*, 1984). The preference for the chemically complex membrane–water interface is suggestive of a role for the LLP sequences as membrane anchors. The (partial) insertion of the LLP sequences into the interfacial region would have a physical influence on the local membrane environment through introduction of local curvature stress as well as by affecting the lateral pressure profile in the membrane interior (Booth *et al.*, 2001; Cantor, 1997b, 1999; Tillman & Cascio, 2003; van den Brink-van der Laan *et al.*, 2004). These physical alterations of the membrane environment by interfacially localized LLP sequences may provide an explanation for the modulation of Env ectodomain conformation induced by point mutations in the LLP (Kalia *et al.*, 2005). While the mutations introduced by Kalia *et al.* maintain the amphipathic potential of the LLP2 region, they alter the net charge of the region from +3 to −1 (Kalia *et al.*, 2005). The resulting overall negative charge of the region may impact its association with the negatively charged inner membrane leaflet by introducing charge–charge incompatibilities with the negatively charged phosphatidylserine headgroups. Altered association (i.e. reduced/abrogated association) of LLP2 with the inner leaflet of the membrane would lead to a change in the local lateral pressure profile of the membrane, which in turn could lead to conformational changes in the protein by altering the arrangement of the transmembrane helices (Cantor, 1997a, 1999, 2002; Marsh, 2007; Tillman & Cascio, 2003; van den Brink-van der Laan *et al.*, 2004).

Arginine conservation relative to lysine in the CTT is another area that invites further consideration. Studies demonstrating a transient exposure of LLP2 sequences during the cell–cell fusion process are suggestive of a mechanism to prefer arginine conservation relative to replacement with lysine. Arginine-rich peptides have been demonstrated to cross cellular membranes, while peptides with lysine instead of arginine do not (Futaki, 2005; Mitchell *et al.*, 2000; Tung & Weissleder, 2003). In addition, arginine-rich peptides have been demonstrated to deliver soluble proteins into the cytoplasm of live cells (Inomata *et al.*, 2009). The apparent traversing of the membrane by LLP2 during the fusion process would seem, then, to require the presence of arginine.

Implications for the overall observed conservation of arginine in the CTT become more interesting in the context of a potentially dynamic CTT topology. Published studies suggest that the topology of the CTT apparently can be distinct between the cellular and virion surface (‘can be’ as a function of the fact that published cellular data does not preclude the existence of virion-like CTT in the cell). This discovery of a cellular topology that is distinct from the virion topology is suggestive of either an alternative, stable topology that is apparently functionally irrelevant to the virion, or a dynamic rearrangement of the topology from the extracellular (lumenal or exposed) state to the internally localized (intracytoplasmic) form.

For the sake of argument, if CTT-exposed Env undergoes dynamic rearrangement by ‘flipping’ sides of the membrane (crossing the hydrocarbon interior in the process) to reach a different topology as was demonstrated for LLP2 (Lu *et al.*, 2008), the presence of arginine relative to lysine would be energetically preferred according to the biological hydrophobicity scale (Hessa *et al.*, 2005a). A movement of arginine-rich sequences across a membrane when captive as part of a larger protein complex (as opposed to soluble peptide fragments) is perhaps best demonstrated in the voltage-sensor paddle of the potassium-gated ion channel KvAP, where the arginine-rich S4 helix (with four arginine residues) is proposed to cross the membrane hydrocarbon interior in response to changes in the membrane electrical field (Jiang *et al.*, 2003). Interestingly, this same helical segment has demonstrated potential to insert into the membrane as a transmembrane helix in spite of the four arginine residues (Hessa *et al.*, 2005b).

Overall, the data summarized in this review demonstrate an intriguing evolution of the prevailing perspective on HIV CTT sequences from a general consensus of being unnecessary and unimportant to the virus, to the identification of numerous diverse functional motifs and roles that influence overall Env structural and critical functional properties. It is intriguing to consider that these CTT-mediated functions may be further investigated to reveal new cellular co-factors that participate in Env trafficking and incorporation into virions and that may serve as novel targets for antiviral drugs. In addition, the data indicating a dynamic topology for the CTT may upon further study reveal heretofore undefined functions associated with the selectively arginine-rich, amphipathic helical sequences that are characteristic of the CTT domains of all HIV Env species, and of all animal lentiviral species.
